# Repeated Intra-Articular Administration of Platelet-Rich Plasma (PRP) in Temporomandibular Disorders: A Clinical Case Series

**DOI:** 10.3390/jcm11154281

**Published:** 2022-07-22

**Authors:** Maciej Sikora, Marcin Sielski, Maciej Chęciński, Zuzanna Nowak, Barbara Czerwińska-Niezabitowska, Dariusz Chlubek

**Affiliations:** 1 Department of Maxillofacial Surgery, Hospital of the Ministry of Interior, Wojska Polskiego 51, 25-375 Kielce, Poland; sikora-maciej@wp.pl (M.S.); marcinsielski@gazeta.pl (M.S.); 2 Department of Biochemistry and Medical Chemistry, Pomeranian Medical University, Powstańców Wielkopolskich 72, 70-111 Szczecin, Poland; 3 Department of Oral Surgery, Preventive Medicine Center, Komorowskiego 12, 30-106 Cracow, Poland; maciej@checinscy.pl; 4 Department of Temporomandibular Disorders, Medical University of Silesia in Katowice, Traugutta 2, 41-800 Zabrze, Poland; zuzannaewanowak33@gmail.com; 5 Specialist Orthodontic Practice: Treatment of Masticatory Organ Dysfunction, Kujawska 19/41, 25-344 Kielce, Poland; baja@netcom.kielce.pl

**Keywords:** temporomandibular joint, temporomandibular disorders, platelet-rich plasma, intracapsular injections

## Abstract

Background: Temporomandibular joint disorders (TMDs) are manifested, inter alia, by pain and limited scope of the mandibular abduction. Among the treatment strategies for these ailments, intra-articular injections of autologous blood preparations, including platelet-rich plasma (PRP), are administered. This prospective case series was aimed at assessing the effectiveness of repeated platelet-rich plasma (PRP) administration to the TMJ cavities in terms of reducing articular pain and increasing the mobility of the mandible. Material and methods: 40 consecutive patients diagnosed with TMJ pain qualified for the case series. The entire treatment program consisted of five PRP administrations and a summary appointment. Regression was analyzed for (1) intensity of spontaneous pain; (2) effectiveness of spontaneous pain relief; (3) mastication efficiency values; (4) painless mandibular abduction; (5) maximum mouth opening. The correlations between the abovementioned variable series were analyzed. Results: The mean spontaneous pain decreased consistently with successive PRP administrations in line with the regression model: −0.4x + 4.2 (R^2^ = 0.98). Articular pain improvement was reported in 71% of joints treated. Improvement in chewing quality at the end of the entire injection cycle was found in 63% of patients. The equations for the linear regression models for painless mandibular abduction (five applications of PRP) and maximum mouth opening (the first four applications of PRP) were x + 34 (R^2^ = 0.89) and 0.6x + 43.6 (R^2^ = 0.96), respectively. Improvement in these domains was found in 78% and 53% of patients, respectively. The strongest correlations were found between pain and chewing efficiency (−0.95), pain and painless mandible abduction (−0.96), and painless mandibular abduction and mastication efficiency (0.94). Conclusion: PRP injections into TMJ cavities should be considered as a low invasive, highly accessible form of treatment for various TMDs causing pain and mandible movement limitation.

## 1. Introduction

### 1.1. Background

Temporomandibular disorders (TMDs) are a common but very general diagnosis [1,2]. According to various diagnosis criteria, TMDs may affect from 7 to 85% of the population [1,2]. According to the ICOP 2020 classification, separate categories of orofacial pain are myofascial pain and pain in the temporomandibular joint (TMJ) [3]. In the case of the latter, pain is often associated with limitation of the mobility of the mandible, which in total translates into lower mastication efficiency and can significantly reduce the quality of life [4].

Paired TMJs, equipped with an extensive system of muscles and ligaments, are responsible for the mobility of the mandible [1,4]. Each TMJ consists of articular surfaces on the temporal bone and the head of the mandible, an articular disc separating these two surfaces, and an articular capsule [4]. Inside the joint capsule there is synovial fluid, the main component of which is hyaluronic acid [4].

The most commonly used methods for treating TMD include pharmacotherapy, physiotherapy, splint therapy, surgery, and intra-articular injections [4,5]. The latter may be rinsing of the joint cavity (called arthrocentesis), intra-articular administration of autogenous blood products (e.g., platelet-rich plasma (PRP) or injectable platelet-rich fibrin (I-PRF)), and drugs, e.g., hyaluronic acid (HA) or corticosteroids [4,5,6,7,8,9,10].

### 1.2. Rationale

Good results of HA supplementation to the inside of the joint cavity may justify intra-articular injections in order to supplement and improve the composition of the synovial fluid [4,9,10,11]. Lavage of the articular cavity also reduces pain and improves mandibular mobility, possibly by reducing inflammatory mediators [4,12]. A single-visit rinsing of the joint cavity followed by injection of HA does not seem to improve the therapeutic effect compared to sole arthrocentesis [13].

The use of autogenous centrifuged blood products has a positive effect on wound healing [14]. PRP and I-PRF contain natural substances that reduce inflammation and are increasingly used both in the treatment of complicated healing and in the reduction of inflammation of intentionally created surgical wounds [14,15]. The studies conducted so far suggest the safety and efficacy of administering PRP to the temporomandibular joint cavity [15]. Nevertheless, the summary of the studies on intra-articular PRP administration does not provide clear evidence of the effectiveness of such a procedure, which implies the need for further studies [13].

### 1.3. Objectives

This prospective case series was aimed at assessing the effectiveness of repeated platelet-rich plasma administration to TMJ cavities in terms of reducing articular pain and increasing the mobility of the mandible.

## 2. Materials and Methods

### 2.1. Case Series Design

Participants in the case series were administered PRP obtained from their own blood into the cavities of the temporomandibular joints in accordance with modern medical indications. The regional bioethics committee approved the case series program. In accordance with the consent of the committee, there was no placebo group or any other control. The report was designed based on the STROBE protocol and checklist [16].

### 2.2. Setting

The case series program was conducted in 2020–2021 at the Department of Maxillofacial Surgery, Hospital of the Ministry of Interior, Kielce, Poland. The qualification period covered two full months from the beginning of August to the end of September 2020. In total, the entire program consisted of 6 medical appointments including 5 therapeutic appointments and a summary appointment. At 5 therapeutic visits, the surgeon (M. Sie.) administered PRP to one or both TMJs, according to prior qualification. At the summary appointment, the patient was examined by an orthodontics specialist (B. C.-N.). The therapy was carried out according to an individual schedule for each patient. The intervals between injections, for organizational reasons, ranged from 7 to 10 days. The summary visit was carried out about a month (±7 days) after the last PRP administration. After this time, each of the patients, in accordance with the assumptions of the program approved by the bioethics committee, could return to other analgesic therapies including pharmacotherapy, physiotherapy, and splint therapy. Therefore, it was not possible to conduct further follow-up visits to assess the effect of intra-articular injections alone.

### 2.3. Intervention

Surgical management at therapeutic visits consisted of (1) disinfecting the skin of the forearm with an alcohol-based Kodan Tincture Forte Colorless (Schülke & Mayr GmbH, Norderstedt, Germany); (2) collecting 8 mL of peripheral venous blood from the elbow flexion; (3) centrifugation of the collected blood to PRP (160 rpm, 0.22 rcf, 5 min); (4) disinfecting the skin of the preaural area with the agent as above; (5) injecting 0.4 mL of the obtained PRP into the upper portion of TMJ according to the protocol described in our previous paper [4].

### 2.4. Participants

Consecutive patients referred to the Maxillofacial Surgery Clinic for intra-articular PRP injections were qualified for the case series. All patients were referred by orthodontics specialists who stated indications for treatment with the use of PRP. The orthodontists diagnosed all patients in within the 3rd subsection of the 1st edition of the ICOP classification, i.e., TMDs [3]. The program enrolled both patients for whom administration of PRP was to be the primary treatment and those for whom other therapeutic methods did not bring the expected results. In the second case, discontinuation of the current therapy was another requirement for inclusion in the program. Completing a full course of treatment within the time frames specified above was the criterion for including the patient in the analysis. It was permissible to use painkillers as an emergency aid in severe pain in consultation with a team of researchers. The inclusion and exclusion criteria are presented in Table 1.

### 2.5. Variables and Data Sources

In order to characterize the case series, data on the sex and age of the patients were obtained in the course of a medical interview. Each of the patients specified how long they had been experiencing soreness in the temporomandibular joint or joints. Medical data from the referral were collected on the diagnosis and the qualification of each joint in each patient for injection. At each of the 6 visits, the patient quantified the current pain on visual analog scales (VAS) for the right and left TMJs as well as the overall mastication performance immediately before the intervention. This scale took integer values from 0 to 10. At each of the medical visits, the range of painless mandibular abduction and nonsupported manually maximum mouth opening were examined. Each time, 3 successive measurements were performed in order to calculate an arithmetical mean.

### 2.6. Bias

The evaluation of the indications and results of the therapy was separated from the individual stages of the treatment. Referral for PRP injections was considered by the orthodontist from our research team (B. C.-N.). This orthodontist confirmed the qualification for injections and ruled out general contraindications. Then, the surgeon (M. Sie.) ruled out local contraindications and administered PRP without knowing the details of the diagnostic process and indications for injection. Finally, the orthodontist (B. C.-N.), who intentionally did not attend individual therapeutic visits, examined the patient and summarized the results of the therapy. The data were analyzed by the authors who deliberately did not participate in the diagnosis, qualification, therapy, or its summary (M. Sik., M. C., and Z. N.).

### 2.7. Case Series Size

Due to the wide discrepancy in epidemiological data regarding the incidence of TMJ dysfunction and pain, it was difficult to estimate the desired sample size. The available budget allowed for the implementation of a full treatment program in 40 patients. Therefore, it was assumed that the case series would be continued until full therapeutic cycles were achieved in 40 consecutive patients.

### 2.8. Quantitative Variables

The characteristics of the case series included the quantitative variables of age, duration of ailments, and the number of joints qualified for treatment in each patient. Subjective assessment of spontaneous pain (*p_n_*) gave 1 value for each visit of each patient, i.e., 6 values per patient (*p*_0_ − *p*_5_). On their basis, during subsequent visits (*n*), the effectiveness (*p_en_*) of the therapy was assessed, expressed by the formula:

*p_en_* = *p*_0_ − *p_n_*

where *p_e_*_5_ expressed the overall pain relief effectiveness.

The mastication efficiency (*m_n_*) was determined analogously on the same VAS at each visit (*m*_0_ − *m*_5_). The increase in mastication efficiency (*m_en_*) was determined by the formula:

*m_en_* = *m_n_* − *m*_0_

where *m_e_*_5_ expressed the overall increase in mastication efficiency.

For maximum painless mandibular abduction (*a_n_*) and maximum mouth opening (*o_n_*), 3 consecutive measurements were made for each patient at each appointment. The mean values of these 3 measurements were taken as quantitative variables, thus giving one *a_n_* value and one *o_n_* value per one visit of one patient, i.e., *a*_0_ − *a*_5_ and *o*_0_ − *o*_5_ per patient. The effectiveness of the therapy in relation to the maximum painless abduction (*a_en_*) of the mandible and the maximum opening of the mouth (*o_en_*) was calculated as follows:

*a_en_* = *a_n_* − *a*_0_

*o_en_* = *o_n_* − *o*_0_

where *a_e_*_5_ and *o_en_* expressed the overall increases in painless mandibular abduction and maximum mouth opening, respectively.

### 2.9. Statistical Method

Regression was analyzed for the (1) intensity of spontaneous pain (*p_n_*); (2) effectiveness of spontaneous pain relief (*p_en_*); (3) mastication efficiency values (*m_n_*); (4) painless mandibular abduction (*a_n_*); (5) maximum mouth opening (*o_n_*). The correlations between the variable series *p*_0–5_, *m*_0–5_, *a*_0–5_, and *o*_0–5_ were analyzed. In the case of missing data, the closest value of a given variable was used, e.g., missing or illegible information on the amplitude of mandibular abduction at the last appointment was supplemented with the value from the penultimate visit. Data analysis was performed using the OriginLab software (OriginLab Corporation, Northampton, MA, USA) and Google Sheets (Google LLC, Mountain View, CA, USA).

## 3. Results

### 3.1. Participants

Forty-two patients were referred for treatment under the research program. We confirmed the indications for PRP injections in 41 of them (the indications for treatment subsided before the initiation of therapy in one patient). One of the patients who started the therapy did not complete it. This patient withdrew from the research program after two bilateral intra-articular PRP administrations due to the ineffectiveness of its pain relief. Initial diagnosis indicated serous TMJ inflammation as manifested by chronic pain. Pain values in this patient were 5 and 8 before the first injection, 6 and 9 before the second, and 6 and 10 after week 1 on the right and left VAS, respectively. Due to the ineffectiveness of the therapy, the patient returned to the referring orthodontist in order to change the treatment strategy. Thus, 40 patients met the criteria for inclusion in the analysis.

### 3.2. Descriptive Data

The group of 40 patients consisted of 36 women and 4 men. The age of the participants ranged from 14 to 78 years. The median age was 33, and the mean was 37.5. Patients were diagnosed in stages II, III, and IV on the Wilkes scale: 28 (70.0%), 7 (17.5%), and 5 (12.5%) of them, respectively. Three patients did not report how long they had suffered from TMDs. Among the remaining 37 participants, the duration of the symptoms ranged from approximately 1 to 30 years, with the median being 4 years and the mean 6 years and 5 months. In 30 patients, both joints were qualified for treatment, and in 10 only one of the TMJs. Thirty-three right and thirty-seven left joints were treated, for a total of seventy joints treated with PRP injections.

Due to an oversight by the investigators, seven patients did not subjectively assess the severity of joint pain and chewing efficiency at the last visit. In all cases, the data from the previous five visits were complete. In accordance with the adopted methodology, the data from the fifth visit were used each time as the values for the sixth visit. In three patients, the pain-free and maximum jaw abduction values were not correctly measured at the last visit. Similarly, in line with the adopted assumptions, the data from the penultimate visit were used. Three patients did not report symptom duration, and their responses were not taken into account in the pooled analysis of this variable. One patient did not report the severity of joint pain on the second visit; thus, data from this patient’s first visit was copied.

### 3.3. Outcome Data

The variables defined on the basis of the VAS, i.e., spontaneous pain and mastication efficiency, took 11 predetermined values that were integers ranging from 0 to 10. The mean mandibular abduction determined on the basis of three consecutive measurements was determined in millimeters and ranged from 15.0 to 54.3. The lowest of these extreme values was recorded on the first visit and the highest on the last visit. Similarly, the maximum mouth opening ranged from 23.0 to 57.3 mm. As with painless abduction, the lowest value was measured at the first visit and the highest after the end of therapy.

### 3.4. Main Results

#### 3.4.1. Spontaneous Pain

**Figure 1:**
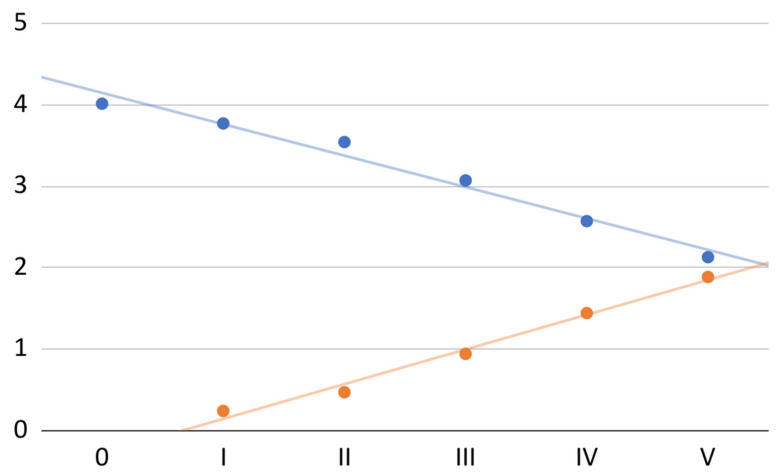
Mean spontaneous pain (blue) and mean effectiveness in pain relief (orange) on the VAS at individual visits.

The mean of subjective spontaneous pain calculated from the values for the 70 treated joints decreased consistently with successive PRP administrations (total decrease of 47%; Figure 1). The trendline expressing the linear regression model (*R*^2^ = 0.98) can, in this case, be described by the equation:

−0.4*x* + 4.2

The same figure also shows the effectiveness of the therapy in relieving subjective pain (Figure 1). Again, the mean values for the individual joints treated were used. The equation of the linear regression model (*R*^2^ = 0.99) took the form:

0.4*x* − 0.3

**Figure A1:**
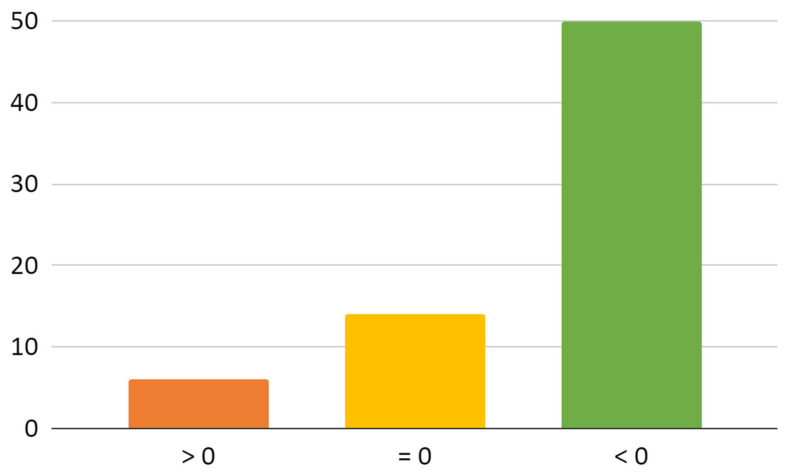
Number of joints with increasing pain (>0), unchanged pain (=0), and pain reduction (<0) based on differences in VAS scores during treatment.

The actual improvement, expressed as the difference in pain scores at the end and start of therapy, was reported by the patients relative to 50 of the 70 joints treated (Figure A1). In the remaining cases there was no improvement (14 joints), or the pain was worse (6 joints). The severity of pain in one case was scored as 1 point, in four cases as 2 points and in one case as 3 points on the VAS.

The case with the greatest increase in pain in the course of therapy (by 3 points on the VAS) can probably be explained by a measurement error. According to the adopted test method, the reference pain value was the one recorded before the first administration of PRP, in this case 5 points. However, the analysis of medical records showed that the previous (not taken into account in our case series) value of pain from this joint was nine. Such a large discrepancy may indicate an unreliable assessment of the patient or an error in understanding the content of the order. It should also be added that in the same patient a decrease in pain by 5 points in the case of the other joint was shown.

#### 3.4.2. Mastication

**Figure 2:**
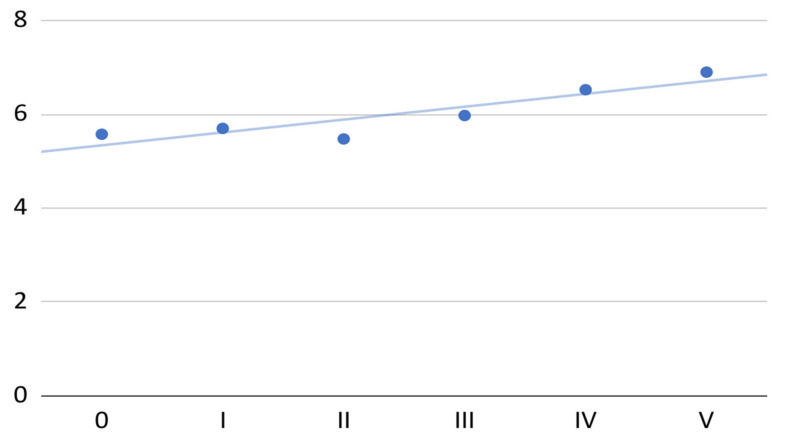
Mean mastication efficiency on the VAS at individual visits.

The mean of subjectively assessed VAS chewing performance in the treated 40 patients increased with the successive administrations of PRP (total increase of 24%; Figure 2). After the second administration of PRP, the mean chewing performance decreased to a value close to the initial value; then, the constant increase in this variable was maintained. The regression analysis showed that it was possible to approximately fit the linear model (*R*^2^ = 0.8) expressed by the equation to the increase in the mean chewing efficiency:

0.3*x* + 5.3

**Figure A2:**
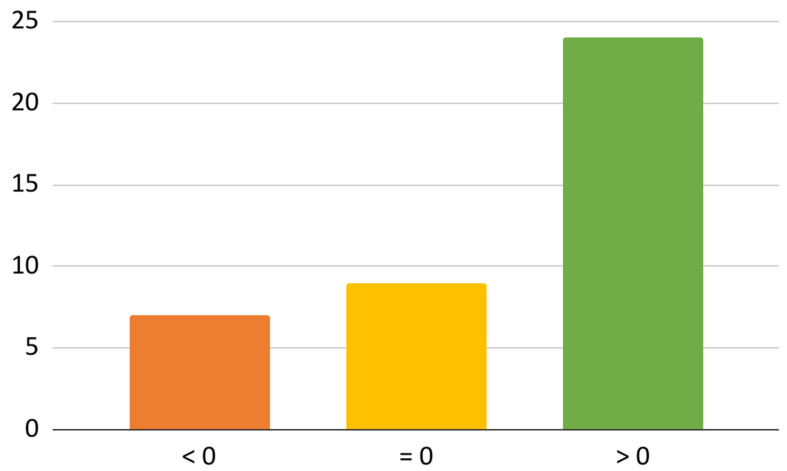
Number of patients who experienced mastication efficiency worsening (<0), mastication efficiency was unchanged (=0), and mastication efficiency was improved (>0) based on differences in VAS scores throughout the course of treatment.

Mastication efficiency as a result of the performed PRP therapy improved in 25 out of 40 patients (Figure A2). In 9 subjects, the difference between the final and initial subjective assessment of chewing efficiency was 0, indicating no change in this range, of which it was between 6 and 10 in seven, 5 in one and 3 VAS in one. A decrease in mastication efficiency was noted in 6 people and it took values down to −4 points. In the patient with the greatest decrease in chewing efficiency, both joints were treated, pain was reduced on both sides (by 3 and 2 points), the scope of painless mandibular abduction increased by approximately 2 mm and the maximum opening of the mouth by 4 mm. Therefore, it can be assumed that a subjective assessment of such a strong decrease in mastication efficiency may result from other reasons or the imperfection of the measurement method.

#### 3.4.3. Painless Mandible Abduction

**Figure 3:**
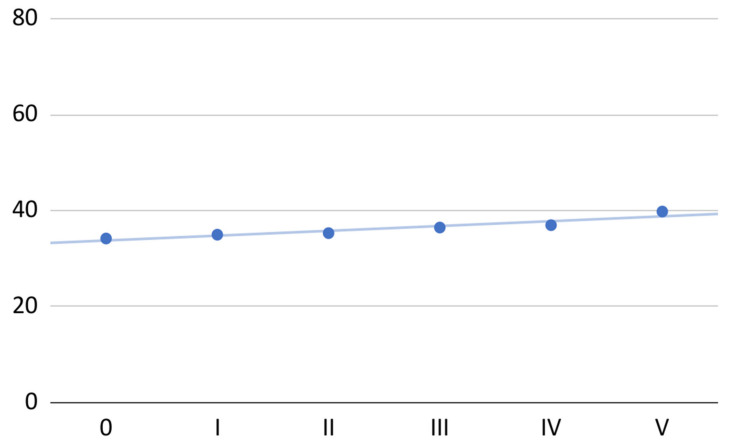
Mean painless mandible abduction at individual visits.

The mean range of painless mandibular abduction for the 40 patients gradually improved over the course of the therapy (total increase of 16%; Figure 3). The improvement was, on average, 1 mm for each PRP administration. An approximate linear regression model (*R*^2^ = 0.89), in this case, can be described by the following equation:

*x* + 34

**Figure A3:**
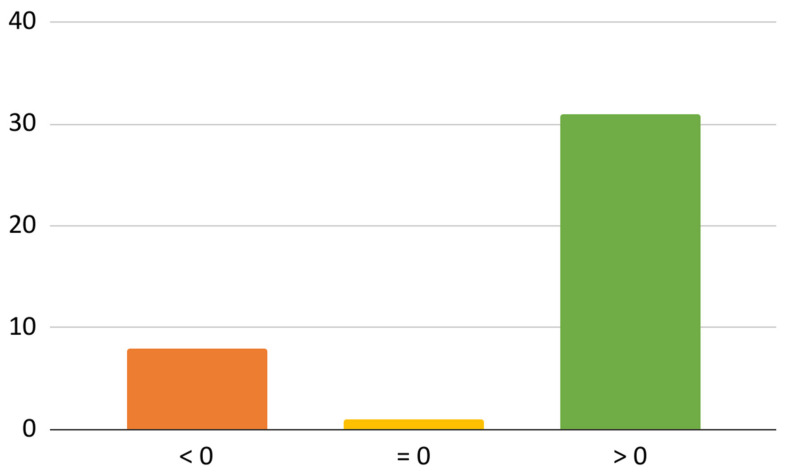
The number of patients who decreased the extent of painless mandibular abduction (<0), the extent of painless mandibular abduction did not change (=0), and the extent increased (<0) based on differences in treatment outcomes.

The extent of painless mandibular abduction increased in 31 out of 40 patients (Figure A3). In one patient, this range, averaged to a tenth of a millimeter from three measurements with an accuracy of 1 mm, did not change over the course of the therapy (it slightly fluctuated during the treatment). In the remaining eight patients, painless-free mandibular abduction decreased, in seven patients by no more than 3 mm and in one by 5 mm. One joint was treated in this patient, and the pain decreased and chewing efficiency increased. The scope of painless mandibular abduction in this patient was approximately 5 mm greater than at each of the subsequent visits, i.e., it decreased by approximately 5 mm from the time of the first administration of PRP and then remained at a lower level. The values of the recorded maximum mouth opening had a similar pattern, also decreasing by 5 mm in relation to the first measurement, with the difference that on the third visit, the maximum mouth opening returned to the initial value (and decreased again later). We could not find an explanation for such a pattern of painless and maximum mandibular abduction, and a measurement error cannot be ruled out here.

#### 3.4.4. Maximum Mouth Opening

**Figure 4:**
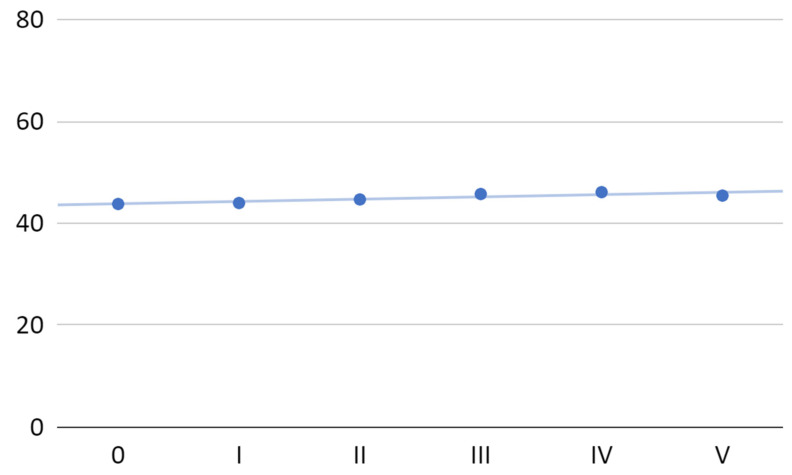
Mean maximum mouth opening at individual visits.

The mean maximum mouth opening gradually increased in 40 patients by the four first PRP injections (total increase by 4%) and decreased after the fifth administration (Figure 4). The overall mean increase in maximal mandibular abduction was 1.6 mm after a series of five PRP administrations. The maximum mean value of the gain (after the fourth administration of PRP) was 2.3 mm. The following approximate linear regression model (*R*^2^ = 0.77) was fitted to all mean values of maximum mandibular abduction:

0.4*x* + 43.8

For the first four administrations of PRP, the linear regression model (*R*^2^ = 0.96) was more accurate and can be described by the equation:

0.6*x* + 43.6

**Figure A4:**
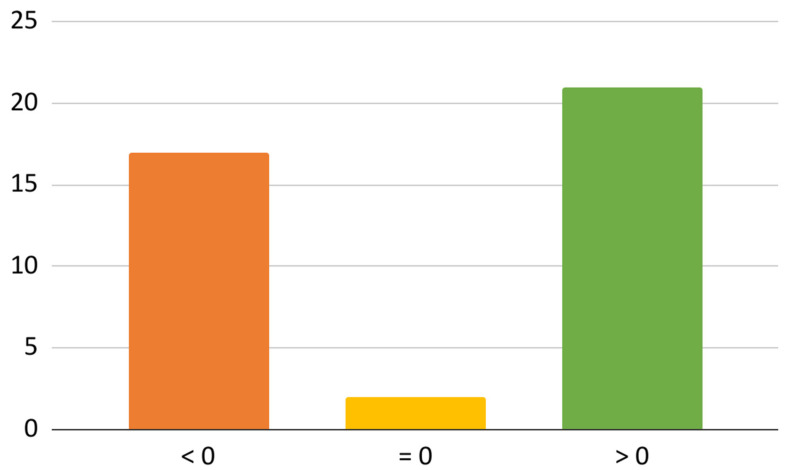
The number of patients who decreased the extent of maximum mouth opening (<0), the extent of maximum mouth opening did not change (=0), and the extent increased (<0) based on differences in treatment outcomes.

The range of maximal mandibular abduction increased after the entire series of PRP injections in 21 out of 40 patients (Figure A4). In the other two patients, this range was equal to a tenth of a millimeter before and after treatment (the means of three consecutive measurements were compared each time). The remaining 17 patients experienced a reduction in maximum mouth opening, and in 11 of these cases the deterioration did not exceed 3 mm. In six cases where a decrease in the maximum abduction range by more than 3 mm was observed, the initial values of the mandibular abduction were greater than 40 mm. The patient, whose maximum mandibular abduction was the most limited, by 8.7 mm in the other parameters (i.e., pain intensity, chewing quality, and painless abduction), showed neither improvement nor deterioration (the final values were very similar to the initial values). Maximum abduction in this patient remained approximately constant up to the four PRP injections and decreased by approximately 10 mm after the fifth injection.

### 3.5. Other Analyses

The analysis of the correlation of mean values of pain (*p*), mastication effectiveness (*m*), painless mandibular abduction (*a*), and maximum mouth opening (*o*) at six medical appointments showed a number of relationships (Table A1). The strongest negative correlations were found between decreasing pain and increasing chewing efficiency and the increasing range of painless abduction. Almost equally strong but positively correlated were the extent of painless mandibular abduction and mastication efficiency. Maximum mouth opening correlated the weakest with other variables, but these correlations were also classified as strong.

## 4. Discussion

### 4.1. Key Results

Results of this case series show that repeated intra-articular administrations of PRP reduced pain and increased mandibular mobility. There was a negative, quite strong correlation (−0.66) between the initial joint pain and the effectiveness in this domain of intra-articular PRP administration applied five times. In the analyzed cases, the average pain reduction on the 11 point VAS was 0.4 per each administration of PRP. In the course of the case series program, the average dynamics of the decrease in pain value did not decrease, which may suggest that the continuation of the therapy could be effective in the context of the entire case series. In 42 examined joints, pain after five injections was less than the average pain after three and four injections, which is presumably a group of potential beneficiaries of continued treatment. In 13 joints, the value of pain calculated in this way increased, which may mean that the limit for repeated PRP administrations was exceeded, and constant values were achieved for 15 joints. A large number of injections per joint (five repetitions) allows for a preliminary drawing of the assumption that the number of injections may be dependent on the resolution of pain and pain-related ailments (i.e., chewing quality and painless abduction of the mandible), which is an individual matter for each patient.

There was quite a weak correlation (−0.36) between the initial subjective assessment of mastication and the effectiveness of its improvement. The negative value of this variable suggests that, on average, the worse the patient assessed his chewing before starting treatment, the better the effect. Analogous calculations for painless (−0.44) and maximum (−0.49) mandibular abduction showed moderate negative correlation. Therefore, it should be assumed that the change in the mobility of the mandible in the course of PRP administration into TMJs cavities depends to some extent on the initial values of the variables. This relationship is clearer in the physical examination than in the subjective assessment of chewing. Most of the patients in the case series improved their chewing efficiency (63%) and maximum jaw abduction without pain (76%). This confirms the analgesic effectiveness of the PRP intra-articular administration. Approximately half of the subjects (53%) experienced an increase in the maximal range of mouth opening, suggesting that there is a potential for PRP to overcome mechanical obstacles to abduction, possibly in the inflammation reduction mechanism. The discrepancy between the number of patients with increased mandibular mobility and the corresponding value without pain (25% of patients) additionally emphasizes the analgesic effect of the therapy.

### 4.2. Limitations

The sources of potential bias or imprecision of our case series lie mostly in the absence of a control group, the short follow up, and small group of patients. The group was composed of patients with TMJ complaints, who required treatment and decided to participate in the project provided that they would be treated. For this reason, already in the assumptions of the project, the possibility of comparing the tested method with therapies expected to be less effective or a placebo was rejected. Injecting the healthy side as a control in treated patients was not chosen for ethical reasons. The small size of the case series and the lack of a control group were also caused by the financial limitations of patients and the lack of funding for the project. Therefore, it cannot be ruled out that future randomized controlled trials will show similar results for placebo or other injectable groups. Further randomized controlled trials are required to better control the study confounders and provide the deep analyses comparing clinical outcomes after TMJ injections with different solutions or medications. The observation period was forced due to the assumption that after the study all of the patients would return to their initial therapies. The variables of the assessment of mastication efficiency and mandible mobility did not allow for an assessment for individual joints but for individual patients. This means that they were analyzed irrespective of whether the application was one-sided or two-sided. Only the variables concerning articular pain were interpretable for each joint separately.

### 4.3. Interpretation

The golden standard in treatment of painful TMDs with limited mouth opening has not been set yet. There is still an ongoing search for an effective, predictable and fast-acting method. Therefore, conducting more clinical trials, as well as drawing specific conclusions, is essential for achieving the objective summary that could lead clinicians in their work.

The therapy turned out to be the most effective in decreasing the overall spontaneous pain. This was also shown in recent studies investigating the application of PRP injections into TMJ cavities. Hegab et al. stated pain and mandible mobility improvements after PRP injections alone in treating TMJ osteoarthritis [17]. However, they highlighted that the treatment showed the best results in long-term observations, meaning from 6 to 12 months [17]. Similar results were obtained by Al-Delayme et al., who achieved pain reduction and improvement of the extent of maximal mouth opening in patients with disc displacement without reduction [18]. A subsequent study by Cömert Kiliç et al. also showed the satisfactory effects of PRP therapy; however, the results indicate no superiority of its use over the HA application [6]. Additionally, it seems important to also highlight the validity of conducting few treatment sessions, as the obtained results showed fluctuations around the initial rounds that afterwards stabilized and indicated clearer improvement in all parameters. In another study, Cömert Kiliç et al. presented similar results, as they described significant improvements in general pain complaint rates, masticatory efficiency, joint sounds, interincisal opening, and lateral motion after multiple PRP administrations [8]. It should be noted here that the analgesic efficacy of intra-articular PRP injections is based on the correct identification of joint pain and distinguishing it from ailments of myofascial origin, which can also be treated but with other methods [1,4,19,20,21]. Limits in the mobility of the mandible suitable for injection therapy should, in turn, be differentiated from mechanical obstacles resulting from fractures with blockage of the fragments, misplacement of the osteosynthetic material, and the consequences of injuries including ankylosis [22,23,24,25].

Patients with various TMDs constitute 31% of the adult population according to a meta-analysis from 2021 by Valesan et al. [26]. They most often present with disturbing pain and mandible movement limitations. Such ailments disrupt everyday life which was shown in different questionnaire studies investigating the quality of life (QoL) of those patients [27,28]. The treatment of intraarticular disorders, such as osteoarthritis, disc displacements, or degenerative joint diseases, is not straightforward, and reports indicate insignificant or very low improvement in QoL [27]. The difference is especially visible when we compare the questionnaires of those patients with answers of the patients suffering from maxillofacial fractures whose recovery is usually smooth and their answers clearly indicate the improvement in QoL within 3 months after the procedures [29]. Prolonging ailments and poor treatment effects contribute to lack of satisfaction with life that might lead to mental disorders and further consequences such as developing alcoholism, losing a job, or worsening interpersonal relationships. For this reason, a fast acting and effective method of reducing pain and movement limitations is essential for maintaining general public health. Intra-articular injections, including administration of PRP, can in this context be regarded as an emergency treatment of TMJ pain and a preventive measure against a deteriorating decline in the quality of life [28,29,30].

Results obtained in this case series are in line with the most recent literature summaries in a form of meta-analyses and/or systematic reviews in the discussed field [31,32,33,34,35,36]. However, in depth comparison of the research available is not straightforward due to the lack of a standardized protocol of intracapsular injections treatment. The mostly used protocols include administering PRP alone, conducting an arthrocentesis or arthroscopy followed by PRP injections or administration of platelet-derived growth factors (PDGF) with or without arthroscopy/arthrocentesis. The leading method seems to be PRP administration immediately after arthrocentesis or arthroscopy which promotes better healing after those procedures [30]. Despite the differences the vast majority of the research highlights the superiority of PRP treatment in any of the above protocols. An obstacle in comparing our work with similar studies is the fact that patients qualified for this case series had various initial diagnoses so comparing our results with clinical trials investigating the results in selected, homogenous groups, e.g., patients with osteoarthritis can be ambiguous. Nevertheless, overall, there was enough evidence to classify the intraarticular injections into TMJ as effective treatment. Within those procedures PRP injections most often provide the best results or at minimum are as good as their alternatives [15,31,32,34,37,38,39]. Another scrupulous analysis of the subject was performed by Derwich et al. in their systematic review on osteoarthritis treatment with intraarticular injections [13]. Among 16 analyzed studies, 5 investigated the effectiveness of application of blood products. Four of them stated no significant differences between examined groups regarding maximum mouth opening; however, one authored by Hegab et al. claimed improving that parameter within 12 months follow up [17]. In the case series of the authors of this article, the standard parameter of maximum mouth opening was distinguished from pain-free opening, and a more pronounced improvement in the values of the latter was observed. The work of Hegab et al. pointed out that the PRP injections brought noticeably better results in MMO and pain reduction than HA. That is consistent, regarding pain control, with the findings of Fernández- Ferro et al. and Toameh et al.; however, they did not notice an improvement in MMO [40,41]. Similar results presented by Gokçe Kutuk et al. claim the superiority of PRP over HA and CS in the treatment of pain in TMJ areas [39]. Pihut et al., in their work from 2020, claimed improvement in pain as well as MMO for both PRP and HA in the treatment of disc displacement without reduction, after the manual disc reposition [38]. Likewise, a report by Pihut et al., from 2014, showed a significant improvement in pain after administration of PRP in the treatment of various TMDs manifesting with pain in the stomatognathic system [37].

The precise mechanism underlying the actions of PRP in improving pain and MMO remains unclear. However, it is known that major growth factors and growth factor families from PRP, such as tissue growth factor-β (TGF-β), insulin-like growth factor 1 (IGF- 1), bone morphogenetic proteins (BMP), platelet-derived growth factor (PDGF), vascular endothelial growth factor (VEGF), epidermal growth factor (EGF), fibroblast growth factor (FGF), and hepatocyte growth factor (HGF), take part in the process of OA healing [42,43,44]. The detailed composition of blood products results not only from the properties of the patient’s blood but also from the method of preparation; hence, apart from PRP used for injection into TMJs cavities, there are also other substances including I-PRF (injectable platelet-rich fibrin) and PRGF (plasma-rich in growth factors) [45,46,47]. Various growth factors play a role in the proliferation and differentiation of chondrocytes, chondrocyte mitosis and extracellular matrix synthesis, and vascular structure formation and regeneration, which are the basis for cartilage regeneration [43]. Concentrated blood products play an important role in maintaining tissue homeostasis and regulation of the inflammation and coagulation processes [43]. Moreover, there are reports confirming that PRP enhances type II collagen and endogenous HA production [43,48,49,50]. Therefore, its positive effect on symptom reduction lies mostly in its regenerative potential as well as its contribution to better lubrication of the joint structures.

PRP is an injection agent that is not manufactured but has to be prepared individually from the patient’s own blood sample. Obtaining PRP from autologous blood has the advantage that the raw material for its production is available to every patient and it is a free resource. Furthermore, this ensures a significantly low risk of allergic reactions or adverse effects caused by the injectate. However, it also contributes to extending the duration of the procedure as well as requiring the use of additional equipment, such as a centrifuge, while alternatives, such as HA, are simply sold in the form of a formulation in vials. Moreover, it should be borne in mind that PRP, as a blood product, is as good as the substrate from which it was prepared, which should be taken into account in poorly nourished patients, those on an unconventional diet, or those suffering from autoimmune diseases.

## 5. Conclusions

According to our research and the literature analysis, PRP injections into TMJ cavities should be considered as a low invasive, highly accessible form of treatment for various TMDs causing pain and mandible movement limitation. Treatment with five administrations of PRP had a more pronounced immediate analgesic effect (improvement in 71% of joints) than in the domain of maximizing maximum mouth opening (improvement in 53% of patients). Improvements were seen in the majority of patients in the subjective chewing performance and pain-free abduction domains directly related to joint pain in 63% and 76% of patients, respectively. In the case series, each subsequent administration of PRP reduced pain on the VAS (0–10) by 0.4, increased chewing efficiency on the VAS (0–10) by 0.3, and increased pain-free abduction by approximately 1 mm. Further research is required, particularly investigating larger groups of patients with longer follow-up periods.

## Figures and Tables

**Table 1:** Inclusion and exclusion criteria for the research program.

Stage	Inclusion Criteria	Exclusion Criteria
Treatment	A referral by an orthodontics specialist for the administration of PRP to one or both cavities of the TMJs. Diagnosis of temporomandibular joint pain attributed to arthritis, disc displacement, or degenerative joint disease.	Acute cases, withdrawal of indications, or presence of contraindications to PRP treatment, i.e., in particular, platelet function disorders, fibrinogen deficiency and anticoagulation treatment as well as local contraindications such as abscess, inflammation, or tumor of the skin, connective tissue, or bone at the puncture site.
Data analysis	Undergoing the entire treatment program, i.e., 5 injection visits every 7–10 days and a summary visit after a month. The inclusion criterion was the presence at the abovementioned visits, regardless of the medical qualification for each subsequent injection.	Undertaking another treatment for pain in the TMJs or mandibular mobility during a research program.

PRP–platelet-rich plasma; TMJs–temporomandibular joints.

## Data Availability

The study data may be available upon request.

## Appendix A

**Table A1:** Table of correlation between the analyzed variables. Description in the text.

	*p*	*m*	*a*	*o*
*p*	X			
*m*	−0.95	X		
*a*	−0.96	0.94	X	
*o*	−0.84	0.71	0.69	X
